# A consensus linkage map of lentil based on DArT markers from three RIL mapping populations

**DOI:** 10.1371/journal.pone.0191375

**Published:** 2018-01-19

**Authors:** Duygu Ates, Secil Aldemir, Ahmad Alsaleh, Semih Erdogmus, Seda Nemli, Abdullah Kahriman, Hakan Ozkan, Albert Vandenberg, Bahattin Tanyolac

**Affiliations:** 1 Department of Bioengineering, Faculty of Engineering, Ege University, Bornova, Izmir, Turkey; 2 Department of Field Crops, Faculty of Agriculture, Cukurova University, Adana, Turkey; 3 Department of Bieoengineering and Genetics, Gumushane University, Gumushane, Turkey; 4 Department of Field Crops, Faculty of Agriculture, Harran University, Sanlı Urfa, Turkey; 5 Crop Development Centre, University of Saskatchewan, Saskatoon, Saskatchewan, Canada; National Bureau of Plant Genetic Resources, INDIA

## Abstract

**Background:**

Lentil (*Lens culinaris* ssp. *culinaris* Medikus) is a diploid (2n = 2x = 14), self-pollinating grain legume with a haploid genome size of about 4 Gbp and is grown throughout the world with current annual production of 4.9 million tonnes.

**Materials and methods:**

A consensus map of lentil (*Lens culinaris* ssp. *culinaris* Medikus) was constructed using three different lentils recombinant inbred line (RIL) populations, including “CDC Redberry” x “ILL7502” (LR8), “ILL8006” x “CDC Milestone” (LR11) and “PI320937” x “Eston” (LR39).

**Results:**

The lentil consensus map was composed of 9,793 DArT markers, covered a total of 977.47 cM with an average distance of 0.10 cM between adjacent markers and constructed 7 linkage groups representing 7 chromosomes of the lentil genome. The consensus map had no gap larger than 12.67 cM and only 5 gaps were found to be between 12.67 cM and 6.0 cM (on LG3 and LG4). The localization of the SNP markers on the lentil consensus map were in general consistent with their localization on the three individual genetic linkage maps and the lentil consensus map has longer map length, higher marker density and shorter average distance between the adjacent markers compared to the component linkage maps.

**Conclusion:**

This high-density consensus map could provide insight into the lentil genome. The consensus map could also help to construct a physical map using a Bacterial Artificial Chromosome library and map based cloning studies. Sequence information of DArT may help localization of orientation scaffolds from Next Generation Sequencing data.

## Introduction

Lentil (*Lens culinaris* ssp. *culinaris* Medikus) is a diploid (2n = 2x = 14), self-pollinating grain legume with a haploid genome size of about 4 Gbp [1]. It is grown throughout the world, and Turkey is the fourth most important lentil producing country (365.000 tons annually) after Canada, Australia, and USA [2]. Average annual lentil production of world is now approaching 6 Mt and per capita consumption of lentil has been increasing faster than human population growth [3]. Lentil is a very important global crop for the human diet since it is an affordable source of carbohydrates (53.9–63.1%), proteins (20.4–30.9%), minerals (1.78–3.1%), oil (0.70–2.0%), trace elements and fiber [4–6]. Therefore, consumption of lentil by humans ensures achievement of the recommended daily nutritional balance and plays a significant role in alleviating malnutrition and micro-nutrient deficiencies [7].

Genetic linkage map construction has become a necessary tool for molecular genetics and plant breeding programs [8]. The availability of large numbers of molecular markers and large mapping populations are the first step for the construction of genetic linkage maps. These maps have served many purposes in basic and applied research. They have become a key tool for physical mapping of genomes. High density linkage maps have direct implementation in breeding researches such as marker-assisted selection (MAS) since they ensure that any gene of interest will be tightly linked to a molecular marker. Such tight linkages can be utilized for MAS of essential genes in breeding programs [9, 10]. The latest applications of high density genetic linkage maps are for orienting and anchoring scaffolds arising from the genome sequence data [11]. Repetitive DNA sequences always populate plant genomes and these can be impossible to resolve when only short reads are available. High density linkage mapping could help to place the sequences in the right orientation [12].

To date, several genetic linkage maps have been constructed from lentil mapping populations. Table 1 presents a summary of these previous lentil linkage maps. The marker density of these maps (change from 34 to 543 markers) is not concentrated enough to meet the requirements for the above applications. On the other hand, length (in cM) of previous linkage maps were very long despite less DNA marker content (such as 3.843 cM with 199 marker [13]; 2.172 cM with 161 marker [14]).

**Table 1:** Information of previous lentil linkage maps.

Reference	Mapping Population	No. of Individuals	No. of markers	Type of Markers	No. of LGs	Map Length (cM)	Average Distance Between Markers (cM)
[15]	F_2_	66	34	RFLP, isozyme, morphological marker	9	333.0	9.8
[16]	RIL	86	177	RAPD, AFLP, RFLP, morphological marker	7	1,073.0	6.1
[17]	F_2_	150	114	RAPD, ISSR, RGA	9	784.1	6.9
[14]	F_2_	113	161	RAPD, ISSR, AFLP, SSR, morphological loci	10	2,172.4	13.5
[9]	RIL	86	283	RAPD, AFLP, SSR	14	751.0	2.6
[18]	F_2_	153	72	RAPD, AFLP, ISSR	11	412.5	5.7
[19]	RIL	108	236	AFLP, SSR, RAPD	12		
[20]	F_2_	113	158	RAPD, ISSR, AFLP, SSR	10	2,392	15.1
[21]	RIL	94	207	AFLP, SSR, RAPD	12	1,868.0	9.0
[8]	RIL	94	166	AFLP, ISSR, RAPD	11	1,396.3	8.4
[22]	RIL	94	196	EST-SSR/ SSR,	11	1,156.4	5.9
[13]	F_2_	114	199	RAPD, SSR, ISSR	11	3,843.4	19.3
[23]	RIL	147	543	SNP, SSR	7	834.7	1.5
[24]	RIL	101	838	GBS	12	538.8	1.0
[25]	RIL	126	216	SSR	7	1,183.7	5.5
[4]	RIL	96	1,784	DArT	7	4,060.6	2.3
[26]	RIL	118	4,177	DArT	7	497.1	0.1

While conventionally a genetic linkage map has been created from a single mapping population, recent efforts to construct linkage maps from multiple mapping populations, termed as “consensus maps”, have gained interest in the scientific community [27]. Construction of a consensus map offers various advantages such as; (i) higher marker density in a single map and better genome coverage, (ii) identification of the position of common markers across mapping populations, (iii) better assignment of LGs to chromosomes, (iv) identification of conserved marker locus positions, (v) detection of chromosomal rearrangements and gene duplication degree (vi) comparison of QTLs or genes of interest across maps and, (vii) creation of a basis for comparing genomes between related species [28–31]. Even though many consensus maps have been constructed in plant species such as maize [32], wheat [33], barley [34], red clover [35], rye [36], soybean [37], chickpea [38], faba bean [39], pearl millet [27] and pea [40], no studies have been reported yet on construction of a consensus map in lentil with a large number of SNPs.

With this in mind, the aim of this study was to construct a consensus map of lentil from 3 different RIL populations based on DArT markers.

## Material and methods

### Mapping populations

Three RIL lentil mapping populations (LR8, LR11 and LR39) developed from 6 parents [“CDC Redberry” x “ILL7502” (LR8) (under review in Genes, Genomes, Genetics Journals), “ILL8006” x “CDC Milestone” (LR11) [26] and “PI320937” x “Eston” (LR39) [4]] were used for the construction of a consensus map. The RILs were developed at the University of Saskatchewan, Canada where resources for genomic and genetic lentil studies have been under development since 2001. All populations were derived by advancing F_1_ plants from the simple cross to the F_2_ generation, and advanced by single seed descent from the F_2_ to F_7_. LR8, LR11 and LR39 populations include 120, 118 and 96 individuals, respectively.

### DNA isolation

DNA was isolated from the young leaves (4–6 week old seedling) of LR8, LR11 and LR39 RILs and their parents. Tissue lyser (Technogen Co., Izmir, Turkey) was used to grind all leaf samples in liquid nitrogen and QIAGEN Isolation Kit (Catalog No. 69181) was applied to extract total genomic DNA from individual RILs and parents.

### DArT marker analyses and construction of consensus map

DArT markers were used from map data of LR8, LR11 [26] and LR39 [4]. For the consensus linkage map, the markers were first analyzed in JoinMap V.4 [41] to detect distortion. The distorted markers were discarded. Remaining markers were used to construct a linkage map. Map distances were estimated using Kosambi Function [42] and LOD score was accepted as 3. Common alleles were integrated using the “combine groups for map integration” module in JoinMap. The loci were ordered using the regression mapping function. Recombination frequency was 0.4. Finally, the lentil consensus map was visualized using the software MapChart [43]. Comparative linkage map was drawn using the Strudel graphical tool that visualizing genetic and physical maps of genomes for comparative purposes [44].

## Results

### Construction of the lentil consensus map

The lentil consensus map was composed of 9,793 DArT markers (Table 2). The largest data sets were from LR8 with a total of 5,372 SNPs followed by LR11 with a total of 2,967 SNPs and LR39 with a total of 1,454 SNPs. These SNP discovery data files were presented as S1, S2, S3 and S4 Excel Files.

**Table 2:** Characteristics of the linkage groups of the lentil consensus map.

Linkage Group	Total Length (cM)	Number of total SNP Markers	Number of SNP Markers (%)	Number of SNP silico Markers from LR8	Number of SNP Markers from LR8	Number of SNP silico Markers from LR11	Number of SNP Markers from LR11	Number of SNP silico Markers from LR39	Number of SNP Markers from LR39	Average Distance Between Markers (cM)
**LG1**	151.78	1,748	17.84	812	290	238	132	206	70	0.09
**LG2**	175.19	1,407	14.36	487	187	403	180	106	44	0.12
**LG3**	167.69	1,214	12.39	660	278	121	53	77	25	0.14
**LG4**	169.19	2,001	20.43	609	224	554	300	225	89	0.08
**LG5**	106.92	1,364	13.92	608	239	244	124	115	34	0.10
**LG6**	117.91	1,190	12.15	324	112	281	120	208	145	0.10
**LG7**	88.79	869	8.87	392	150	158	59	81	29	0.10
**Grand Total**	977.47	9,793		3,892	1,480	1,999	968	1,018	436	**Average:** 0.10

Seven linkage groups were constructed, corresponding to the number of haploid chromosome. All SNPs were distributed almost evenly in 7 linkage groups. The consensus map spanned a total of 977.47 cM with an average distance of 0.10 cM between adjacent markers. While LG4 had the highest number (1,407) of SNPs, LG7 contained the lowest number (869) of SNPs. The longest LG was LG2 (175.19 cM) with a mean distance of 0.12 cM between adjacent markers and the shortest LG was LG7 (88.79 cM) with a mean distance of 0.10 cM between adjacent markers. All linkage group size and mean distance between adjacent markers are presented in Table 2.

Among 7 LGs, LG4 contained the highest marker density (1 marker/0.08 cM) and LG3 had the lowest marker density (1 marker/0.12 cM). In general, marker density was consistent throughout the map. The consensus map had no gap larger than 12.67 cM and only 5 gaps were found to be between 12.67 cM and 6.0 cM (on LG3 and LG4). The largest gap between adjacent markers was 12.67 cM on LG3 between the marker “4088505” (at 43.87 cM position) and “3629574” (at 56.55 cM position) and 9.37 cM on LG4, between the marker “4087364” (at 153.92 cM position) and “4087288” (at 163.30 cM position) (S4 Excel File).

### Comparison of consensus map and component maps

**Fig 1:**
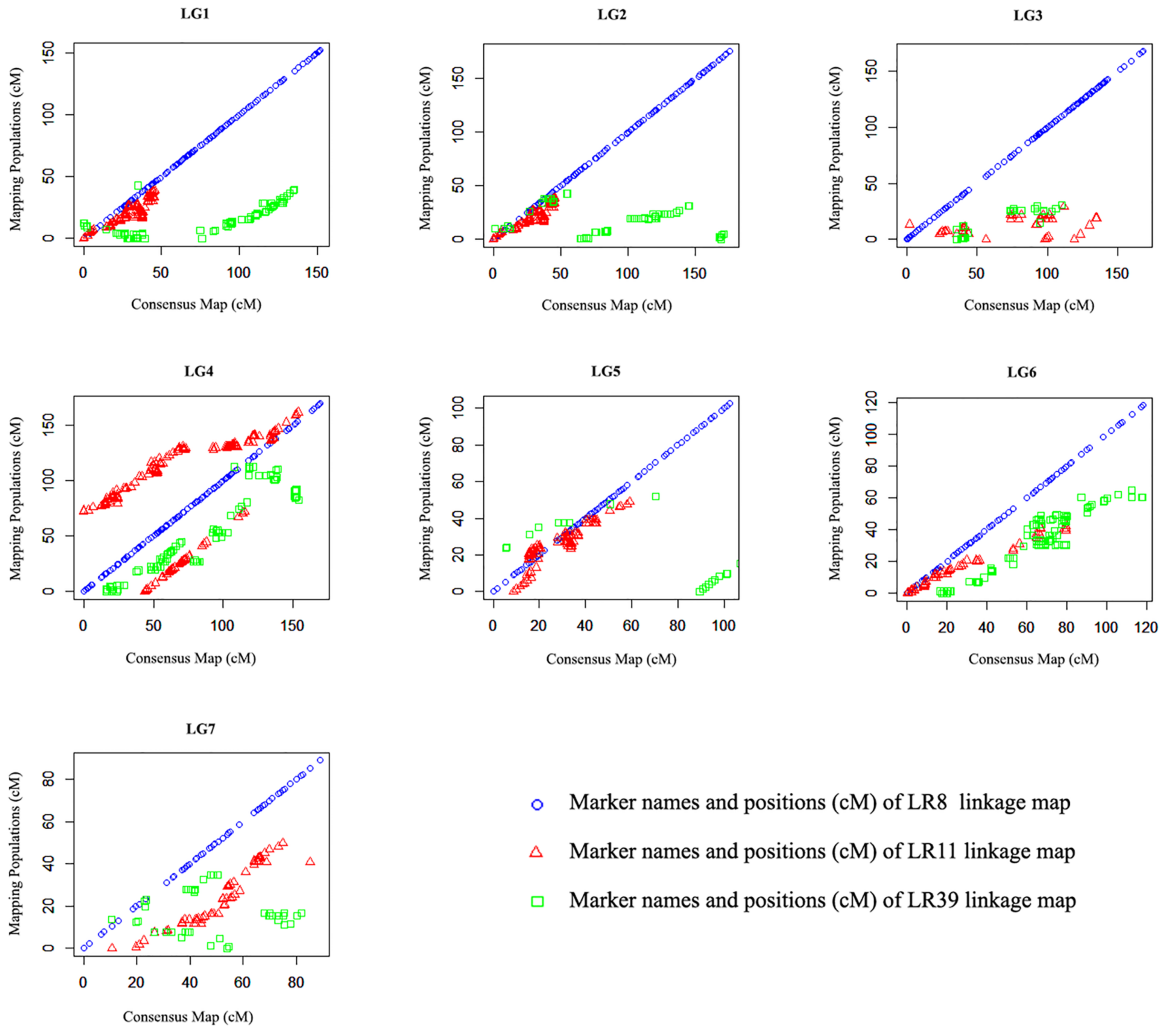
Comparison of marker locations in consensus map and component maps.

**Fig 2:**
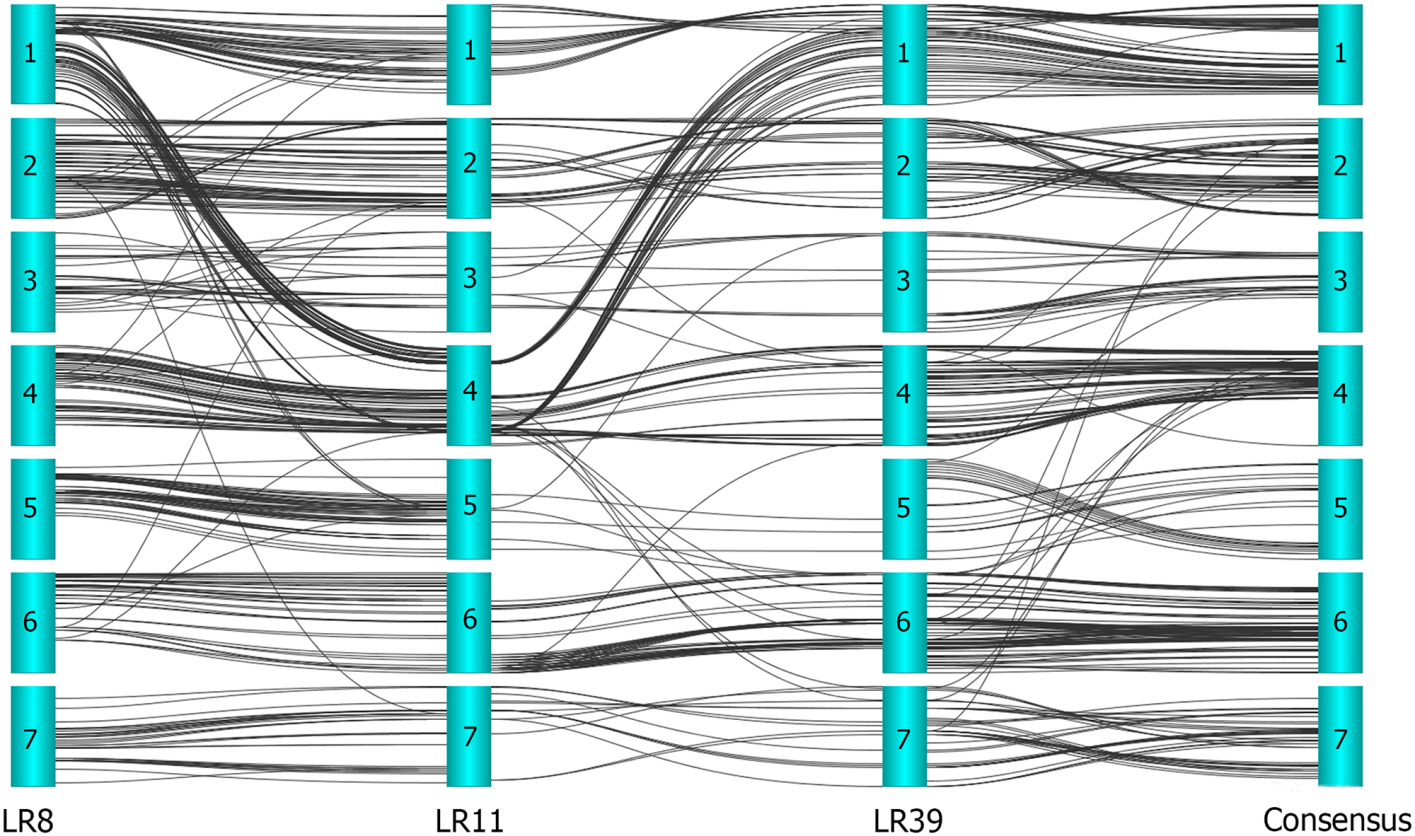
Comparison of linkage groups from the consensus map and component maps.

For interpretation of the quality of the lentil consensus map, the consistency of marker order between the component genetic linkage maps (LR8, LR11 and LR39) and the lentil consensus map were compared. Due to this comparison, the marker locations of the lentil consensus map were plotted against the locations in the component genetic linkage maps separately for each LGs (Figs 1 and 2).

The localization of the SNP markers based on the DArT markers on the lentil consensus map were in general consistent with their localization on the three individual genetic linkage maps of the lentil RIL populations. Locations of SNPs among the component maps and the consensus map showed great parallelism (Fig 2). But localization of some SNPs showed variation between component and consensus map. For example, marker “4091453” mapped at 8.27 cM on LG1 of LR8 linkage map, but it was mapped at the 15.62 cM position on the LG1 of the consensus map. Total map length, number of mapped SNPs, the average distance of adjacent markers of consensus map, and the component maps are shown in (Table 3). The lentil consensus map has longer map length, higher marker density, and shorter average distance between the adjacent markers compared to the component linkage maps (Table 3).

**Table 3:** Characteristics of the lentil consensus map and the component maps.

	Consensus Map				Component Maps											
					LR8 Population				LR11 Population				LR39 Population			
LGs	Length (cM)	Number of total SNP Markers	Number of SNP Markers (%)	Average Distance Between Markers (cM)	Length (cM)	Number of total SNP Markers	Number of SNP Markers (%)	Average Distance Between Markers (cM)	Length (cM)	Number of total SNP Markers	Number of SNP Markers (%)	Average Distance Between Markers (cM)	Length (cM)	Number of total SNP Markers	Number of SNP Markers (%)	Average Distance Between Markers (cM)
**LG1**	**151.78**	**1,748**	**17.8**	**0.09**	**151.8**	**1,102**	**20.5**	**0.13**	**38.9**	**516**	**12.4**	**0.08**	**42.7**	**524**	**21.5**	**0.08**
**LG2**	**175.19**	**1,407**	**14.3**	**0.12**	**175.2**	**676**	**12.5**	**0.25**	**126.5**	**724**	**17.3**	**0.17**	**42.5**	**314**	**12.9**	**0.13**
**LG3**	**167.69**	**1,214**	**12.3**	**0.14**	**167.7**	**940**	**17.4**	**0.17**	**29.0**	**258**	**6.2**	**0.11**	**30.6**	**166**	**6.8**	**0.18**
**LG4**	**169.19**	**2,001**	**20.4**	**0.08**	**169.2**	**835**	**15.5**	**0.20**	**161.5**	**1,224**	**29.3**	**0.13**	**112.3**	**450**	**18.5**	**0.25**
**LG5**	**106.92**	**1,364**	**13.9**	**0.10**	**102.5**	**849**	**15.8**	**0.12**	**48.9**	**593**	**14.2**	**0.08**	**51.8**	**161**	**6.6**	**0.32**
**LG6**	**117.91**	**1,190**	**12.1**	**0.10**	**117.9**	**439**	**8.1**	**0.26**	**42.5**	**523**	**12.5**	**0.08**	**64.8**	**628**	**25.7**	**0.10**
**LG7**	**88.79**	**869**	**8.8**	**0.10**	**88.8**	**544**	**10.1**	**0.16**	**49.8**	**339**	**8.1**	**0.15**	**34.7**	**196**	**8.0**	**0.18**
**Grand Total**	**977.47**	**9,793**		**Average: 0.10**	**973.1**	**5,385**		**Average: 0.18**	**497.1**	**4,177**		**Average: 0.12**	**379.4**	**2,439**		**Average: 0.15**
**Reference**					**(Under review in Genes/Genomes/Genetics Journal)**				[26]				[4]			

## Discussion

In the current study, we constructed the first consensus genetic map for lentil based on DArT markers. Nowadays, there is no standard naming convention for integrated genetic linkage maps [35]. For this reason, an integrated map is alternately termed as consensus, comprehensive, reference, composite, or pooled map depending on the procedure of integration [45]. In this study, we constructed a lentil consensus genetic linkage map using the JoinMap software (V.4.) [41]. This consensus map is based on mean frequencies ratio of recombination and integrated multiple data sets of segregation [46]. The mapped loci positions were mostly quite conserved between the merging map and the component maps, which pointed out that the localization of the loci can be considered as the “consensus” positions. Therefore, in the current study we termed the lentil map we constructed a “consensus map”.

### Mapping populations

To date, several genetic linkage maps have been constructed for lentil, and while in earlier mapping studies, lentil linkage genetic maps were constructed mostly based on F_2_ populations [14, 15, 17], more recently mostly RIL populations were used to construct lentil linkage genetic maps [4, 8, 9, 21, 23, 25]. A mapping RIL population is the result of many recombination generations, ensuring greater chances for separation of linked genes and markers and linkage breakdown [47]. On average, the chance of recombination between tightly linked genes in a RIL mapping population is twice as much as that in a BC_1_ or an F_2_ mapping population [48], therefore allowing a more correct map distance conjecture [49].

In the present study, 3 different lentil RIL mapping populations [LR8, LR11 [26] and LR39 [4]] were used to construct a lentil consensus map. Similarly, RIL mapping populations were used to construct consensus map of other crops such as wheat [33], barley [34], soybean [37], chickpea [38], rye [28], faba bean [39], pearl millet [27] and pea [40].

If the genes are tightly linked, a greater number of individuals in a RIL mapping population are required in order to obtain the accurate gene order with high confidence [50]. The number of individuals in LR8, LR11 and LR39 populations were 120, 96 and 118 respectively. It is clear that, in mapping studies, the use of a population which includes an insufficient number of individuals results in erroneous ordering of fragmentation and loci of the LGs [51].

### Advantage of DArT markers

Recent advances in next generation sequences (NGS) provide high throughput data, which opens the way for the detection of SNP markers. NGS also reduces the cost of SNP detection by using the reduced representation method [52, 53]. Building a consensus map is impossible without common loci present on each chromosome or linkage group [34]. Therefore, increasing the number of common markers in the different lentil populations has great importance. DArT is a recently developed molecular technique used for construction of the lentil maps in the current study. Besides lentil, DArT technology has been used to construct consensus maps of other crops such as barley [54], sorghum [55], rye [28], triticale [56] and rapeseed [46].

DArT markers define polymorphisms via changes of single base-pair (SNPs) at recognition sites of restriction enzymes [28]. SNP polymorphisms account for about 90% of the genetic variation in any organism and are equally distributed among a genome [57]. One advantage of this technology is that it does not require previous sequence information data for the plants to be studied. Another advantage of the DArT markers is that they can generate thousands of markers in a short time at low cost [28].

### Characteristics of lentil consensus map

SNP discovery has received much attention during the last decade due to their distribution throughout genomes and also allows construction of high density linkage maps. High density linkage maps are useful for developing understanding of the structural organization of a genome [58]. In the current study we constructed the first consensus map of the lentil genome, consisting of 9,793 markers (based on DArT markers), covering 977.42 cM and spanning all 7 chromosomes corresponding to the 7 haploid chromosome number of lentil [4] (Table 2). The total length of consensus map (977.42 cM) was similar to the 834.7 cM map reported by Sharpe et al., [23]. The mean average marker density of this consensus map is one marker per 0.1 cM. Previously reported lentil genetic linkage maps consisted of smaller numbers of markers (varied from 34 to 5,385 markers) and longer mean marker density between adjacent markers (chanced from 0.12 to 19.3 cM) compared to the current consensus map [4, 8, 9, 13–17, 21, 23, 25]. Although the current map contained 15 times more markers than the consensus map reported by Sudheesh et al., [59] (689 SNP markers, 2429.6 cM), the length of the current map is ~2.5 times shorter (977.42 cM). The differences of marker density in the lentil linkage maps might be due to use of insufficient numbers of markers and and/or different types of genetic marker systems, poor and/or missing quality data, marker distribution and crossovers in the lentil genome, number of individuals used in a mapping population and preferences for different linkage mapping methodology [39, 60]. A few large gaps (about 12 cM in length) were detected in this study. A total of only 5 large gaps (about 12 cM; on LG3 and LG4) were detected in the consensus map but this could be due to the mapping of markers with gene in a genome that contains largely of repetitive elements and regions of low polymorphism in an intraspecific population [23] and homozygosity of the lentil genome in this specific region. Previously reported lentil linkage maps included numerous numbers of larger gaps than the current consensus map [4, 8, 9, 13–17, 21, 23, 25]. Our results suggest that DArT technology can be useful to fill the genotyping gap between adjacent markers and to construct well saturated lentil consensus maps, thus resulting in better lentil genome coverage [61].

### Comparison of consensus map and component maps

One of the ways to evaluate the quality of the lentil consensus map is to compare the marker order of the consensus map with the marker order of the component maps [54]. The localization of the SNP markers on the lentil consensus map were generally consistent with their localization on the component maps but some marker positions changed on the LG of consensus map according to this marker position on the LG of component maps. A similar situation was reported for a consensus map of triticale and the author noted that on a more global level, the collinearity plots revealed some differences in length among the component maps and the consensus map. In the consensus map, identical pairs of loci resulted in shorter linkage map distances [56]. In addition, such inconsistencies could refiect actual differences in genome organization between mapping populations or they could be attributed to either the effect of small sample size on the estimated gene orders or the differences in local recombination frequencies between populations. Rearrangements of closely linked markers, particularly those located at distal ends of linkage groups, have previously been observed in grape, cotton, and rubber among other plant species [62, 63].

In the current study, despite local inversions (between LR8(LG1) and LR11(LG4) the SNP locus orders were mostly congruent between the consensus and the component maps (Fig 2). While approximately 119 SNP markers were located between 57–116 cM on LG1 in the linkage map of LR8 (pop 1), these markers located between 4–29 cM on LG4 in the linkage map of LR11 (pop 2) (Fig 2). These minor inconsistencies in marker positions are not rare in map integration [37, 56, 64]. However these kinds of inconsistencies could be due to differences in genome organization among mapping populations. A small number of individuals in the mapping populations could affect the calculation of marker order [64]. Local recombination frequencies among mapping populations could also cause this kind of discrepancy [56]. Similarly, closely linked markers rearrangements have previously been reported in grape [62], cotton [63] and rubber [64].

The current lentil consensus map contains higher marker density and shorter average distance between the adjacent markers compared to the component linkage maps constructed to date. In addition, the total map length of the consensus map was longer than the length of the component maps (Table 3). Similar map expansion was reported for the consensus maps of pepper [65] and rapeseed [66]. In our lentil consensus map, expansion was observed on LG5 due to the addition of markers in this LG [8].

## Conclusion

The recent study reports the first consensus map of lentil from DArT markers by merging large sets of mapping data from 3 lentil RIL populations. This consensus map provides the basis for development of available of genetic markers for genome studies such as construction of physical mapping, collinearity analysis, and map-based gene cloning. Since the consensus map contains large numbers of SNPs, it could also be helpful in marker assisted selection studies. Sequence information of SNPs could help localization of appropriate orientation of the scaffolds from Next Generation Sequencing data.

## Supporting information

S1 Excel File SNP discovery raw data from LR8 populations. (XLSX)

S2 Excel File SNP discovery raw data from LR11 populations. (XLSX)

S3 Excel File SNP discovery raw data from LR39 populations. (XLSX)

S4 Excel File SNP discovery raw data of lentil consensus map. (XLSX)
